# Feasibility of microencapsulated phytochemical as disinfectant for inhibition of *Candida albicans* proliferation on denture base produced by digital light processing

**DOI:** 10.1371/journal.pone.0287867

**Published:** 2023-07-12

**Authors:** Ye-Hyeon Jo, Won-Jun Lee, Hyung-In Yoon

**Affiliations:** 1 Dental Research Institute, Seoul National University School of Dentistry, Seoul, Republic of Korea; 2 Department of Prosthodontics, School of Dentistry and Dental Research Institute, Seoul National University, Seoul, Republic of Korea; Medical University of South Carolina, UNITED STATES

## Abstract

**Backgrounds:**

A proper disinfection of denture is vital to prevent a fungal infection. A study on the feasibility of microencapsulated phytochemical as complementary disinfectant and its interaction with effervescent tablet immersion on denture base resin is lacking.

**Objectives:**

The aim of this study was to examine the feasibility of phytochemical-filled microcapsules as disinfectant for the inhibition of *Candida albicans* (*C*. *albicans*) attachment on the denture base produced by digital light processing (DLP).

**Methods:**

54 denture base specimens uniformly mixed with or without 5wt% phytochemical-filled microcapsules were prepared using DLP. Fungal cells were inoculated onto the surfaces of the specimens, which were divided into three different disinfection treatment groups (n = 9): 1) none, 2) sterile tap water immersion for 15 min, and 3) effervescent tablet immersion for 15 min. After each treatment, the biofilm on denture surface was stained with a crystal violet solution to measure the absorbance. The number of fungal colonies was counted as colony-forming units (CFU) per mL. Morphological changes were examined by microscopy. An aligned rank transform analysis of variance was performed to analyze the interaction of presence of microcapsule and disinfection condition, with statistical significance set at P < 0.05.

**Results:**

Both for the absorbance and CFU, there was no significant interaction between the presence of microcapsules and disinfection conditions (P = 0.543 and P = 0.077, respectively). The presence of microcapsules was statistically significant (both P < 0.001), while the effect of disinfection condition was not significant (P = 0.165 and P = 0.189, respectively). Morphological changes in fungi were detected in the groups containing microcapsules, whereas undamaged hyphal structures were found in those without microcapsules, irrespective of disinfection treatments.

**Conclusions:**

The presence of phytochemical-filled microcapsules significantly reduced the adhesion of *C*. *albicans* and inhibited its proliferation on denture surfaces, regardless of disinfection conditions.

## Introduction

Proper care and hygiene of removable dentures and mucosal tissues of the edentulous mouth have an important impact on treatment success and general health, particularly in older people [1–6]. Mechanical cleaning of dentures is an effective measure for routine control of oral biofilms [7]. However, some denture wearers may have difficulty in keeping their dentures clean, particularly older people who fail to keep their dentures clean due to systemically compromised conditions [8]. Without proper maintenance, the surface of removable dentures can easily be colonized by microbial biofilms, causing mucosal lesions or even favoring the development of systemic infections [2–4]. Contaminated dentures are either a direct cause or a contributing factor to mucosal diseases [9]. Denture-related stomatitis, one of the most common forms of oral candidiasis, is an inflammatory fungal infection, involving *Candida albicans* (*C*. *albicans*), which affects approximately 50–75% of otherwise healthy denture wearers [10–14].

Poly (methyl methacrylate) (PMMA) is an acrylic material widely used in denture applications due to its useful attributes, including its processing simplicity, light weight, low cost, esthetic properties, and stability in the oral environment [15, 16]. However, one of its main drawbacks is its poor antimicrobial property, which allows attachment of microorganisms and accumulation of biofilms [17, 18]. Without proper disinfection, the surface of PMMA-based denture can act as a reservoir for microorganisms and may lead to infectious lesions [17]. Recently, three-dimensional (3D) printing has emerged as a promising technology for denture fabrication, with high productivity and accuracy [19, 20]. Jeon et al. reported that 3D-printable resins for denture bases can have antifungal activities by using microencapsulation of phytochemicals [21]. The 3D-printed denture base containing phytoncide-filled microcapsules showed sufficient antifungal activities, inhibiting proliferation of *C*. *albicans*, while satisfying the clinical requirements in terms of mechanical properties and fabrication trueness [22].

As daily maintenance care for removable dentures, occasionally along with mechanical brushing, soaking removable dentures in solutions of chemical disinfectants (i.e., hydrogen peroxide) or effervescent tablets has been reported to be an effective method for controlling biofilm formation or colonization of microbial cells, such as *C*. *albicans* [23–27]. For the people who cannot perform proper mechanical brushing of dentures, effervescent tablets could be helpful in maintaining denture hygiene. However, disinfection with effervescent tablets cannot completely remove biofilms or attached fungal colonies from the denture surface [28, 29]. Incorporation of intrinsic antifungal activity into denture resins, through microencapsulation and 3D printing, could be beneficial to denture wearers with vulnerability to fungal infection.

To our knowledge, no study has examined the feasibility of microencapsulated phytochemical as complementary disinfectant on the denture base, considering the influence of chemical disinfectant such as effervescent tablet immersion. A study on the feasibility of microencapsulated phytochemical as complementary disinfectant and interaction between effervescent tablet and microcapsules in denture base resin in terms of denture disinfection may broaden the knowledge on clinical applicability. Therefore, the aim of this in vitro study was to investigate the relationship between phytochemical-filled microcapsules in 3D-printed denture bases and disinfection conditions in terms of antifungal effect. The null hypothesis was that no statistically significant difference would be detected in terms of the antifungal effect between the 3D-printed denture bases with and without phytochemical-filled microcapsules, regardless of denture disinfection conditions.

## Materials and methods

A disc with a 15-mm diameter and 5-mm thickness was designed as a testing specimen (TinkerCAD, Autodesk, Montreal, QC, Canada). To fabricate the phytochemical-filled microcapsules, a phytoncide oil was extracted from *Pinus densiflora* (Cleandiox Ltd., Gyeonggi-do, Korea) and incorporated into microcapsules using melamine-based polymers, as reported in a previous study [21]. The phytochemical-filled microcapsules were mixed with a 3D-printable denture-base resin (NextDent Denture 3D+, Vertex Dental BV, Soesterberg, Netherlands), at a concentration of 5wt% [22]. A dispersant (DISPERBYK-111, BYK-Chemie, Wesel, Germany) was also added at 20wt% of the mass (g) of microcapsules to the microcapsule-printable resin mixture for uniform dispersion. The disc specimens were produced from this resin mixture by means of a digital light processing-based printer (MAX UV, Asiga, Alexandria, NSW, Australia) equipped with a 385-nm wavelength light source. The layer thickness and the build angle for the 3D printing were set as 50 μm and 0°, i.e., horizontal to the build platform, respectively (Asiga Composer 1.2.12, Asiga, Alexandria, NSW, Australia). As a control, disc specimens were also manufactured from the same 3D-printable resin, but without microcapsules or dispersant, using the aforementioned parameters. All discs were then cleaned in ethanol in a washing machine (Form Wash, Formlabs Inc., Somerville, MA, USA) for 10 min, and the support structures were carefully removed prior to further treatment. The discs were further treated with a curing unit for 10 min for post-polymerization (Cure M U102H, Graphy, Seoul, Korea).

*C*. *albicans* strain ATCC 10231, provided by the Korean Collection for Oral Microorganisms, was cultured in Sabouraud dextrose (SD) broth (Difco^™^, BD, Franklin Lakes, NY, USA) at 37°C for 24 h. The 3D-printed denture base resin discs with and without phytochemical-filled microcapsules were sterilized with ethylene oxide gas and then placed at the bottom of a 12-well culture plate (SPL Life Sciences Co., Pocheon, Korea). In each disc well, 2 mL of *C*. *albicans* suspension (optical density 0.02 at 600 nm, equivalent to 2 × 10^4^ fungal cells per mL) was added and the plates were incubated for 24 h at 37°C. The discs were then washed twice with Dulbecco’s phosphate-buffered saline (DPBS, Welgene, Gyeongsan, Korea), to remove non-adherent fungal cells.

After incubation and biofilm formation, the discs with or without microcapsules were further treated using the following conditions. To simulate the daily maintenance care patterns of denture wearers, three different denture disinfection conditions were designed as follows: 1) no treatment, to mimic the absence of daily disinfection; 2) syringe-filtered sterile tap water (at room temperature) for 15 min, to mimic simplified rinsing with tap water only, with no additional mechanical brushing, and 3) effervescent tablet immersion (Polident Quick, GSK Consumer Healthcare, Dublin, Ireland), to mimic 15-min storage in denture disinfection solution. An effervescent tablet solution was fabricated by soaking an effervescent tablet in 150 mL of syringe-filtered sterile tap water under an ambient atmosphere, as indicated by the manufacturer. Each 3D-printed denture base resin disc with attached fungal cells was then randomly treated using one of three different disinfection conditions, and no additional mechanical brushing was performed. For the second and third conditions, 3 mL of sterile tap water or effervescent tablet solution was added to a well containing each disc, respectively. Thus, a total of 54 denture base resin discs, with or without phytochemical-filled microcapsules were allocated to each disinfection condition group and treated accordingly (n = 9).

After treatment of the discs with different disinfection conditions, the surfaces of three discs per group were rinsed twice with DPBS. One milliliter of crystal violet solution (1% w/v) was added to each well for staining, and the specimens were then allowed to stand at ambient temperature for 10 min. After the crystal violet solution was removed, each disc was transferred to a new 12-well plate. The discs were then washed four times with DPBS. A destaining solution (ethanol: distilled water = 1:1) was added to each well (2 ml). Subsequently, 200 μL of this dissolved crystal violet solution was aliquoted to a new 96-well plate (SPL Life Science co., Pocheon, Seoul) in six replicates. Absorbance was measured at 600 nm using a microplate reader (Epoch2, BioTek, Winooski, VT, USA) to detect the amount of biofilm.

To count fungal cell colonies, three discs per group were placed into conical tubes containing 5 ml of SD broth after treatment with different disinfection conditions. *C*. *albicans* was detached from the disc surfaces by ultrasonication at 40 kHz for 5 min (NXPC-B5020SB; Kodo Technical Research Co. Ltd., Hwaseong, Korea). The sonicated fungal solutions were serially diluted in DPBS, and 10^4^-diluted 10-μL suspensions were seeded onto the SD agar plates in five replicates. The plates were incubated at 37°C under aerobic conditions for 24 h, and the fungal cell colonies were counted for each disc group as colony-forming units per mL (× 10^4^ CFU/mL). For microscopic examination of morphological changes in *C*. *albicans* on each disc after treatment with disinfection conditions, three discs per group were cleansed with DPBS and fixed in 2.5% (v/v) glutaraldehyde (Sigma–Aldrich, St Louis, MO, USA) for 2 h at room temperature. Subsequently, the fixative was aspirated and cleaned with DPBS and then post-fixed in 1% osmium tetra oxide for 30 min. After fixation, samples were dehydrated in a graded series of ethanol solutions. After critical-point drying, the discs were sputter-coated with Pt and microscopically examined using field-emission scanning electron microscopy (FE-SEM, Apreo S, Thermo Fisher Scientific, Waltham, MA, USA) at 10 kV.

After treatment with different disinfection conditions, the viability of adherent *C*. *albicans* on the disc surfaces (n = 3) was also assessed by staining using LIVE/DEAD BacLight Bacterial Viability kit (L7012, ThermoFisher scientific, USA), according to the manufacturer’s instruction. Briefly, equal volume of SYTO 9 dye and propidium iodide were mixed thoroughly and used to stain alive (green fluorescence) and dead fungi (red fluorescence), respectively. After the 15-min incubation, the stained samples were washed twice with DPBS and then observed by confocal laser microscopy (CLSM; LSM700; Carl Zeiss, USA), at a magnification of ×200.

The data of this in vitro study were not normally distributed, according to the Shapiro–Wilk test, and the assumption of homogeneity of variances was violated according to Levene’s test. Therefore, a non-parametric aligned rank transform analysis of variance (ANOVA) test was performed as a factorial ANOVA to analyze the interaction of two factors (presence of microcapsules and denture disinfection condition) and the main effect of each factor. For possible differences between the groups, a post-hoc multiple comparison was performed using the Mann–Whitney U test with Bonferroni correction. All statistical analyses were performed using SPSS version 25 (IBM Corp., Armonk, NY, USA), and statistical significance was set at *P* < 0.05.

## Results

**Fig 1:**
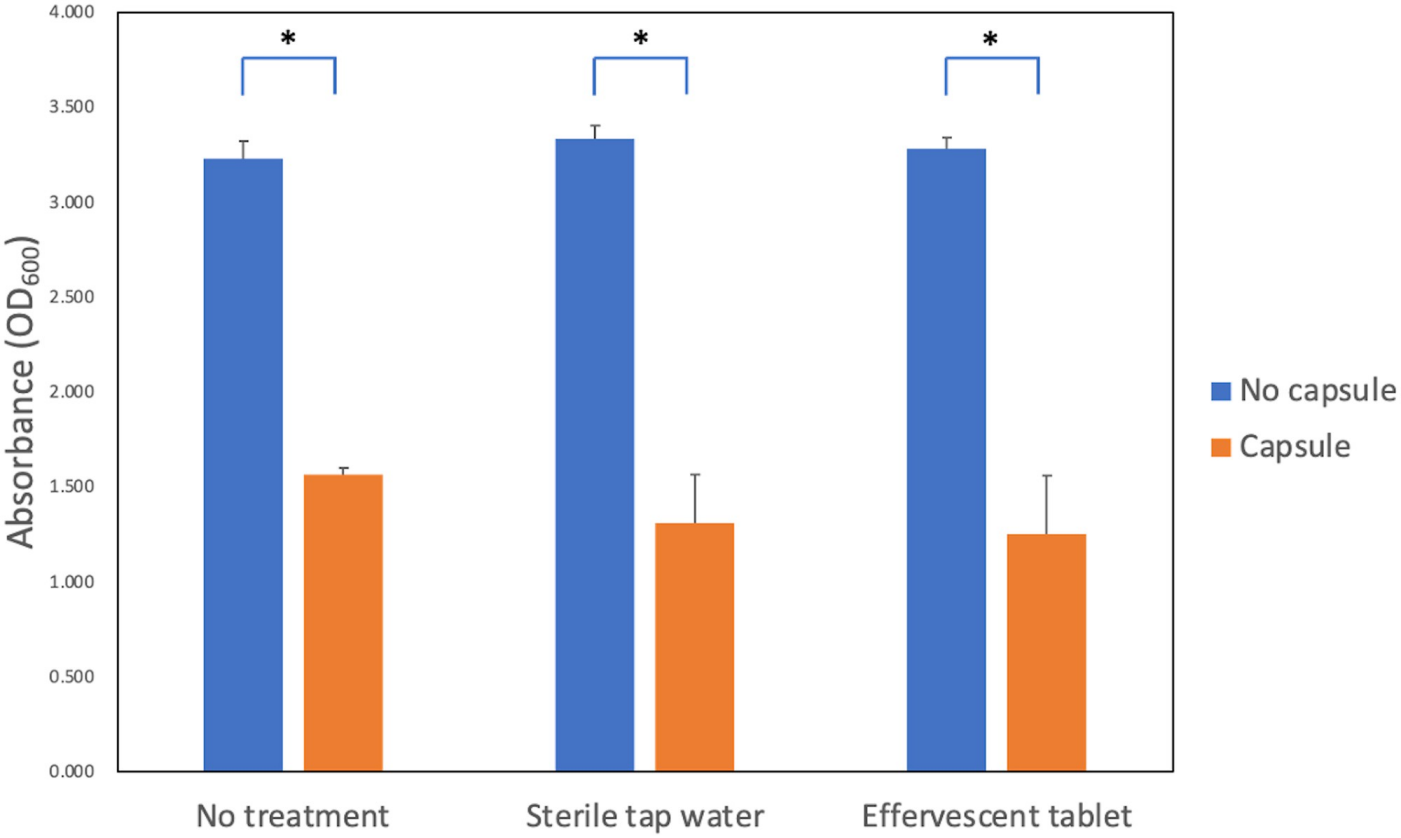
Absorbance of *C*. *albicans* biofilm detached from 3D-printed denture base resin discs. Absorbance (optical density), measured at 600 nm, of *C*. *albicans* detached from 3D-printed denture base resin discs with (capsule) and without phytochemical-filled microcapsules (no capsule), each treated using three different denture cleansing protocols: 1) no treatment, 2) syringe-filtered sterile tap water storage for 15 min, and 3) effervescent tablet immersion for 15 min. Statistically significant difference is marked with an asterisk (*, P < 0.05).

Based on the statistical analysis of the optical density (absorbance) measurements, there was no statistically significant interaction between the presence of phytochemical-filled microcapsules and denture cleaning conditions (p = 0.543). Regarding the main effect, only the presence of microcapsules was significant factor associated with the difference in optical density (p < 0.001), whereas the denture disinfection condition was not (p = 0.165). Based on the optical density measurement, the 3D-printed denture resin discs with microcapsules showed significantly lower optical density than those without microcapsules, regardless of the denture disinfection conditions (Fig 1).

**Fig 2:**
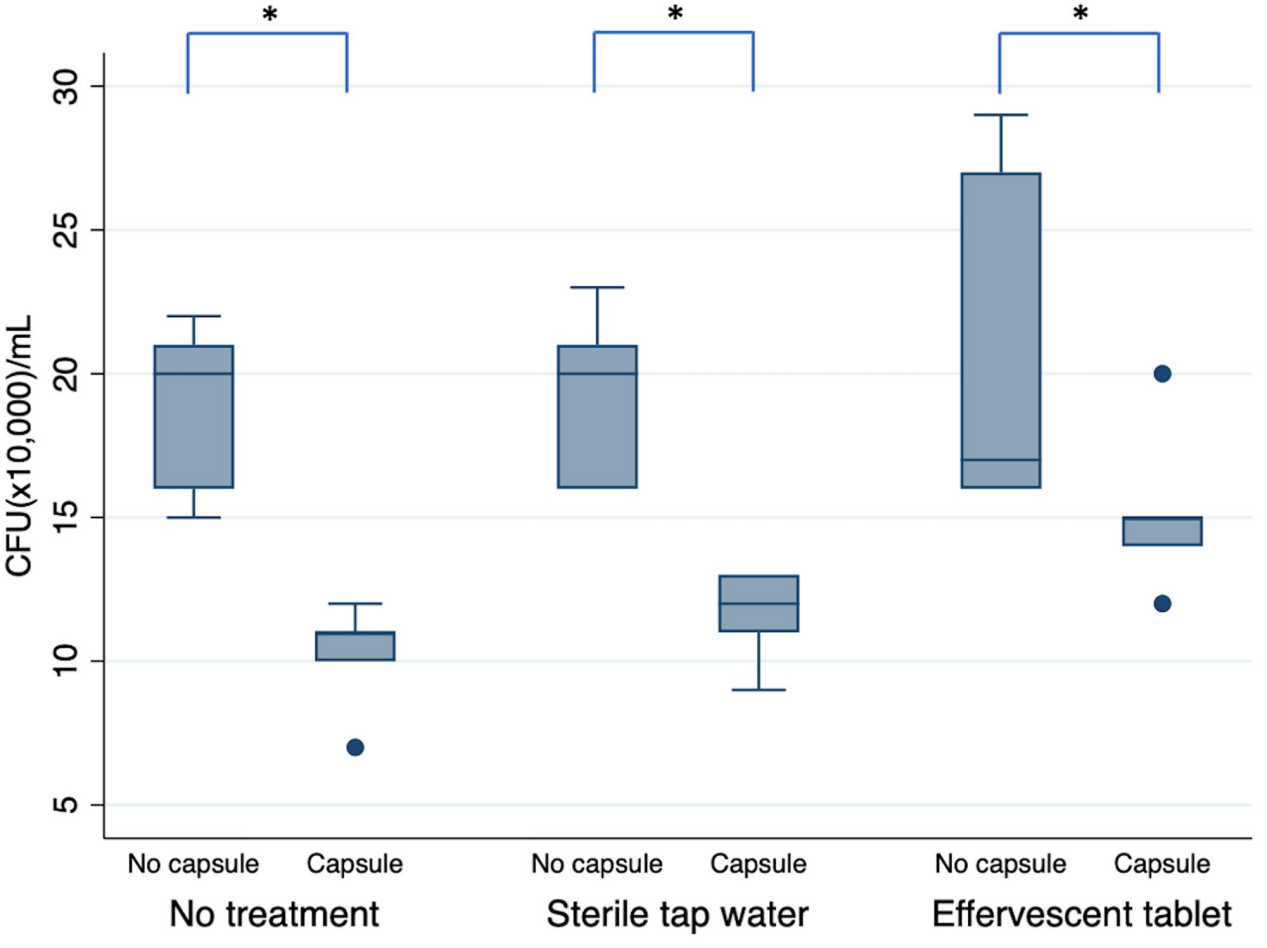
Colony-forming unit per milliliter of *C*. *albicans* detached from 3D-printed denture base resin discs with and without phytochemical-filled microcapsules. Colony-forming unit per milliliter (CFU [×10,000]/mL) of *C*. *albicans* detached from 3D-printed denture base resin discs with (capsule) and without phytochemical-filled microcapsules (no capsule), each treated using three different denture disinfection conditions: 1) no treatment, 2) syringe-filtered sterile tap water storage for 15 min, and 3) effervescent tablet immersion for 15 min. Statistically significant difference is marked with an asterisk (*, P < 0.05).

**Fig 3:**
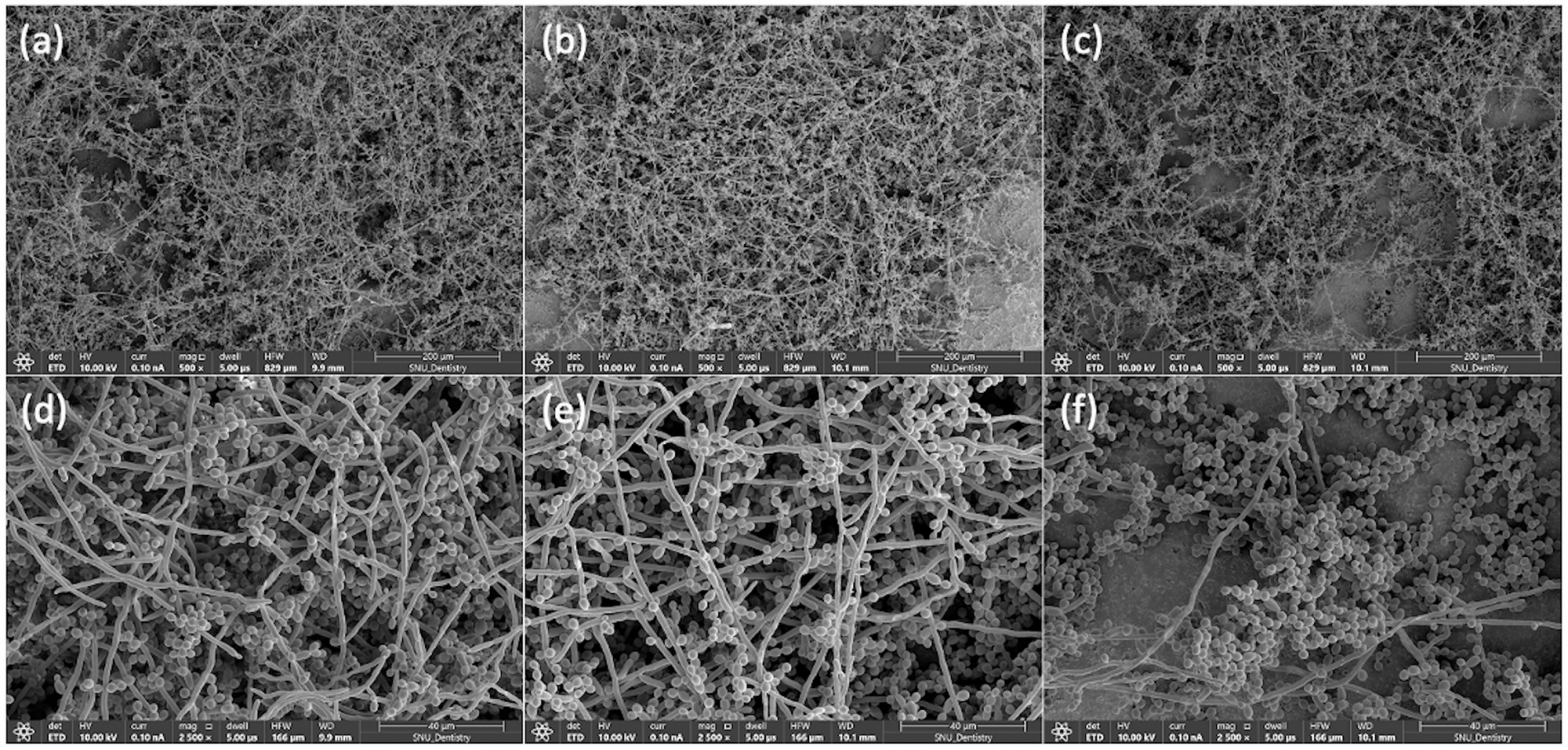
Fungal adhesion on 3D-printed denture base resin discs without phytochemical-filled microcapsules. Representative microscopic images (magnification ×500, upper, and ×2,500, lower) of *C*. *albicans* adhesion on 3D-printed denture base resin discs without phytochemical-filled microcapsules, treated using three different denture disinfection conditions: 1) no treatment ((a) and (d)), 2) syringe-filtered sterile tap water storage for 15 min ((b) and (e)), and 3) effervescent tablet immersion for 15 min ((c) and (f)).

**Fig 4:**
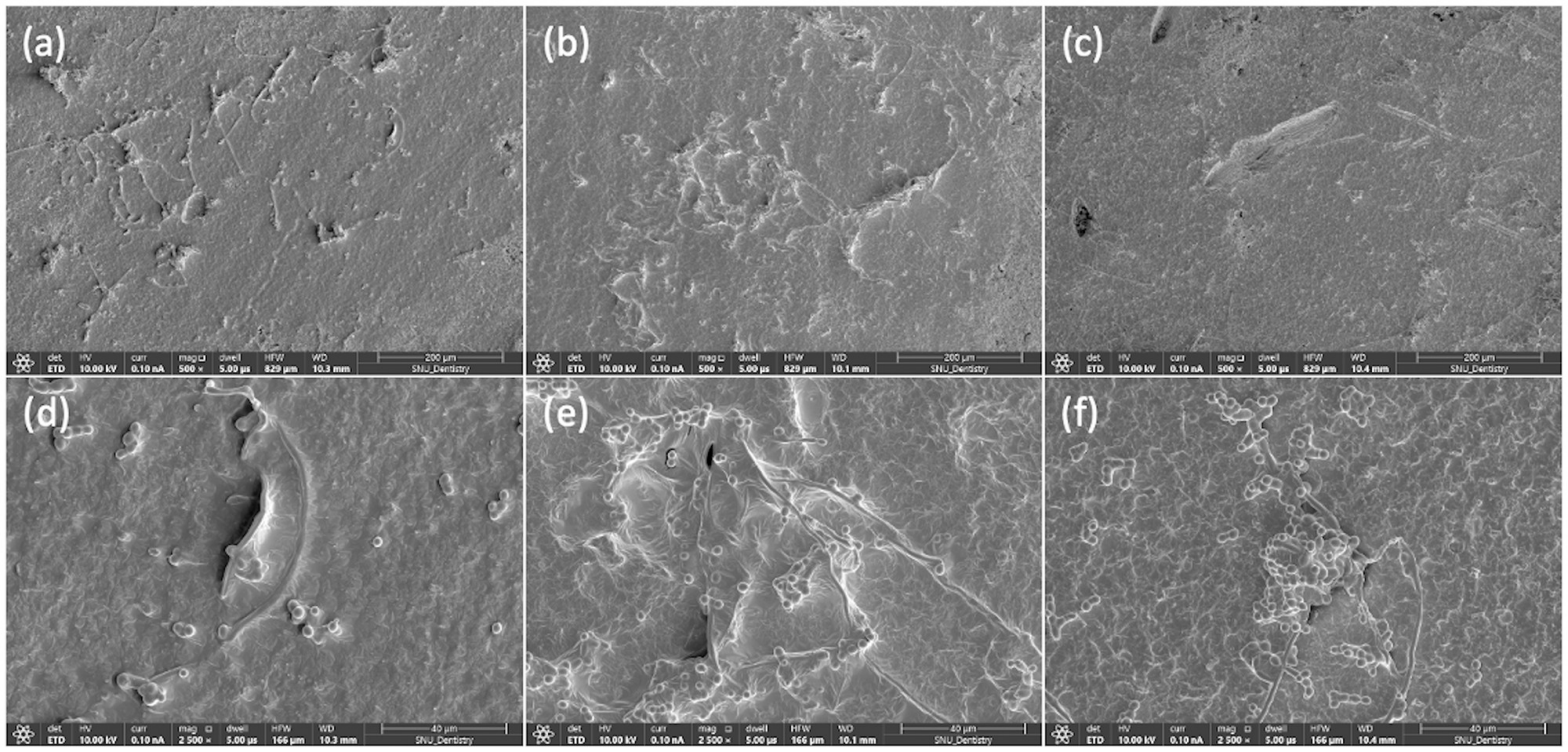
Fungal adhesion on 3D-printed denture base resin discs containing phytochemical-filled microcapsules. Representative microscopic images (magnification ×500, upper, and ×2,500, lower) of *C*. *albicans* adhesion on 3D-printed denture base resin discs containing phytochemical-filled microcapsules, treated using three different denture disinfection conditions: 1) no treatment ((a) and (d)), 2) syringe-filtered sterile tap water storage for 15 min ((b) and (e)), and 3) effervescent tablet immersion for 15 min ((c) and (f)).

Statistical analysis of the fungal colony counting data revealed that the main effect of the presence of microcapsules was statistically significant (p < 0.001), while the effect of the denture disinfection condition was not statistically significant (p = 0.189). No statistically significant interaction was detected between the presence of microcapsules and denture disinfection condition (p = 0.077). Based on the colony counting data, the 3D-printed denture resin discs filled with microcapsules showed significantly fewer fungal cell colonies than those without microcapsules, irrespective of denture disinfection conditions (Fig 2). Microscopic observation revealed that morphological changes in *C*. *albicans*, such as deformed structure or destroyed cell membrane, were clearly detected in the denture-base resin discs with phytochemical-filled microcapsules (Fig 3). For those without microcapsules, numerous fungal hyphal structures, without distinctive morphological changes were found, regardless of the disinfection conditions (Fig 4).

**Fig 5:**
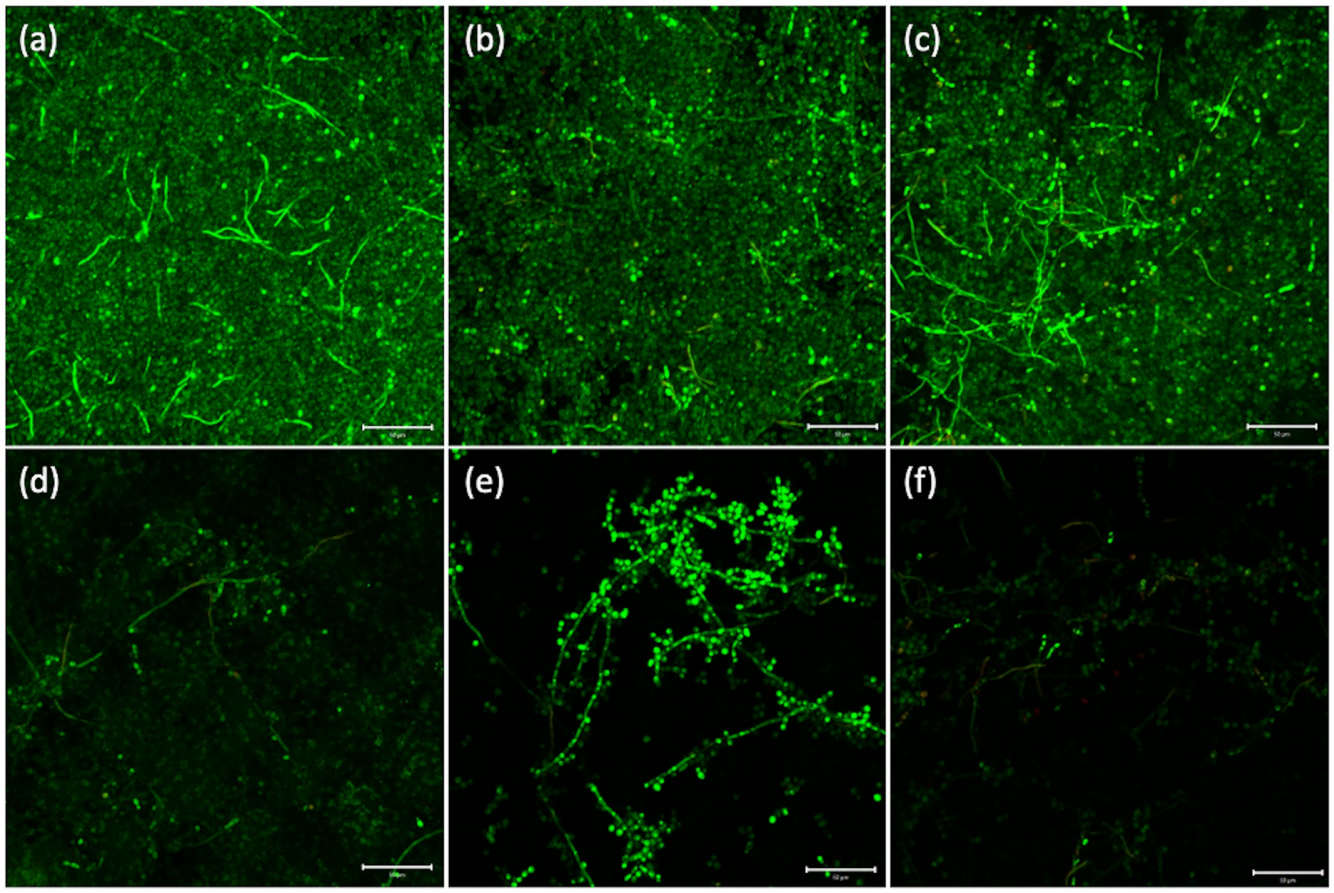
Live/dead staining images of *C*. *albicans* attached on 3D-printed denture base resin discs with and without phytochemical-filled microcapsules. Representative live/dead staining images (magnification ×200) of *C*. *albicans* attached on 3D-printed denture base resin discs with no microcapsules ((a), (b), and (c)), and discs containing phytochemical-filled microcapsules ((d), (e), and (f)), treated by three different denture cleansing protocols; 1) no treatment ((a) and (d)), 2) syringe-filtered sterile tap water storage for 15 min ((b) and (e)), and 3) effervescent tablet immersion for 15 min ((c) and (f)). The live and dead fungal cells exhibited green and red fluorescence, respectively.

In terms of cell viability, the fungal live/dead staining showed distinctive color difference between the 3D-printed denture resin discs with phytochemical-filled microcapsules and those without (Fig 5). Regardless of denture disinfection condition, relatively high number of living fungi, stained with green fluorescence, was detected in the groups with no microcapsules. On the contrary, almost none of living fungi could be found on the discs with phytochemical-filled microcapsules, irrespective of applied condition. With the presence of microcapsules, the effervescent tablet immersion seemed to be slightly more effective in inhibition of fungal cell growth compared with sterile tap water storage.

## Discussion

Based on the findings of this study, the 3D-printed denture resin discs with phytochemical-filled microcapsules showed significantly lower optical density and number of fungal cell colonies than those prepared without the microcapsules, regardless of the applied disinfection condition. Therefore, the null hypothesis of this study was rejected. In addition, denture disinfection method, such as effervescent tablet immersion, was not effective in reducing *C*. *albicans* adhesion or proliferation of colonies on the surface of 3D-printed denture base. The presence of phytochemical-filled microcapsules significantly reduced fungal cell adhesion and inhibited cell proliferation on the surfaces of denture base, which was consistent with previous studies [21, 22]. At a certain concentration of phytochemical-filled microcapsules, the 3D-printed denture base showed significant antifungal activity against *C*. *albicans* for up to 4 weeks, with no cytotoxicity to human gingival fibroblasts [21, 22].

The inhibitory action of effervescent tablets on PMMA-based denture resins, particularly against *C*. *albicans*, has previously been reported [29, 30]. On the surface of the heat-polymerized PMMA denture base resin, a decrease in the adhesion of *C*. *albicans* was more evident in the effervescent tablet solution than in the distilled water storage condition [9]. Fungal cell adhesion decreased after treatment with a microwave-polymerized PMMA denture resin with effervescent tablets [30]. However, the effervescent tablet is not sufficient to eliminate fungal cell colonies, whereas a chemical agent, such as sodium hypochlorite, has been proven to be more effective [29, 30]. Interestingly, both optical density measurements and cell colony counting data revealed that effervescent tablet immersion did not result in significant differences from tap water storage, in terms of inhibiting fungal cell proliferation. In this study, the denture base discs were manufactured by vat polymerization, in contrast to the production methods of specimens in previous studies, which involved heat-polymerization or microwave-assisted techniques [9, 30]. In addition, unlike the current study, in which the surfaces were as-printed without polishing, the samples of previous studies had polished surfaces [9, 30]. Surface roughness has been reported to affect the adhesion of colonies of fungal cells, such as *C*. *albicans* [31]. The biofilm of fungal cell colonies could not be completely removed using the effervescent tablet alone [29, 32]. Inhibition of the proliferation of *C*. *albicans* was also affected by the concentration of the tablet [33]. Differences in the surface roughness between the unpolished, as-printed denture base specimens and the polished specimens fabricated by conventional methods in the previous studies may have affected the fungal cell adhesion differently, and a high concentration of effervescent tablet solution would have been necessary for the removal of *C*. *albicans* in this study.

The characteristics of the denture base materials have been reported to have a significant impact on the adhesion of *C*. *albicans* [34]. Higher adhesion of *C*. *albicans* was been reported in denture bases produced by vat-polymerization than in those fabricated by conventional methods [35]. High adhesion of fungal cell colonies in 3D-printed denture base resin might be associated with the different levels of adsorption of mucin [35]. Furthermore, the increase in the older population is expected to result in an increased number of denture wearers as well as a worse pattern of denture hygiene maintenance [2, 8]. It is difficult to address repeated fungal infections unless the surface of the denture base is appropriately treated [36]. Mechanical cleaning and additional chemical disinfection have been suggested as denture care protocols for the patients with denture stomatitis. However, these methods require voluntary patient cooperation, and mostly can be performed as post-treatment. Since the microencapsulated phytochemical-filled denture base has antifungal activity in itself, it can be a preventive or auxiliary option if the patient’s ability for denture hygiene maintenance is insufficient. Because the antifungal activity of the denture base itself prevents *C*. *albicans* colonization on its surface, it can be an excellent option in cases when mechanical disinfection is difficult [37]. To impart antifungal activity to the denture base, functional bioactive additives such as phytochemical-filled microcapsules, can be mixed with the denture base resin used for 3D printing, which may be an effective manufacturing method to provide customized options for denture wearers who need preventive or treatment care.

There are some limitations of this study. First, the sample size and short duration of analysis may have affected the outcome of the current research. Second, the denture base resin and denture disinfectant were limited to one type of each, more studies on various types of materials regarding the efficacy and their interaction with microcapsules are required. Third, the shape of denture resin specimen was different from clinical denture base, which may have affected the current outcome differently from clinical situation.

## Conclusion

Within the limitations of this in vitro study, the presence of phytochemical-filled microcapsules significantly reduced the adhesion of *C*. *albicans* and inhibited its proliferation on the surfaces of 3D-printed denture base resins, regardless of the disinfection condition applied. Without the help of microencapsulated phytochemicals, denture disinfection treatment such as effervescent tablet immersion alone may not be clearly effective in reducing the adhesion or proliferation of fungal cell colonies on the 3D-printed denture base surface.

## Supporting information

S1 Data (XLSX)

S2 Data (XLSX)
